# Supplementary material for: Depolarization of multidomain ferroelectric materials
Source: Nat Commun. 2019 Jun 11;10:2547. doi: 10.1038/s41467-019-10530-4 (PMC6560217; doi:10.1038/s41467-019-10530-4)
Supplement: Supplementary file 1 — Supplementary Information [file 41467_2019_10530_MOESM1_ESM.pdf]

Supplementary Information

Depolarization of multidomain ferroelectric materials

D. Zhao et al.

Supplementary Note 1. Introduction

In Supplementary Note 2 we discuss suppression of polarization by a dead interface layer or by a finite screening length in the electrodes. We show that in our experiment using thick ferroelectric films these effects can be disregarded. In Supplementary Note 3 we present how the remanent polarization of a ferroelectric capacitor in series with a linear capacitor can graphically be extracted from the quasi-static hysteresis loop of the ferroelectric-only capacitor. We have investigated the depolarization dynamics using a linear capacitor in series with a ferroelectric capacitor. We applied a voltage pulse, high enough to fully polarize the ferroelectric capacitor. Then the applied voltage abruptly dropped to 0 V, and we recorded the transient of the electric displacement, $D(t)$. In Supplementary Note 4 we show that the depolarization transients can be calculated with the Kolmogorov-Avrami-Ishibashi (KAI) formalism, adapted to a time-dependent electric field and Merz law. A good agreement is obtained. In Supplementary Note 5 we show that the depolarization transients exhibit a negative differential capacitance (*NDC*). The value is equal to that of the serial capacitance. In Supplementary Note 6 we determined the temperature dependence of $P_r/\varepsilon_0\varepsilon_{ferro}E_c$ for P(VDF-TrFE). We show that the value is about 15 and independent of temperature between 213 K and 333 K. The constant value implies that at all temperatures the depolarization field, E_{dep}, is similar to the activation field, E_{act}. In Supplementary Note 7 we discuss the physics of intrinsic switching and we reproduce the relation between the intrinsic, thermodynamic coercive field and ferroelectric polarization as theoretically derived in the seminal work of Tagantsev et al. using the Ginzburg-Landau-Devonshire formalism. Subsequently we discuss experimental data on the intrinsic coercive field of $BaTiO_3$ and P(VDF-TrFE).

Supplementary Note 2. Depolarization due to electrode screening and dead layers

In a ferroelectric capacitor the metallic electrodes provide free charges that fully compensate the depolarization field, yielding a zero internal electric field inside the ferroelectric material. When the depolarization field is not fully compensated, the remanent polarization is suppressed. Incomplete compensation can in practice be due to the occurrence of a capacitive dead layer between the electrodes and the ferroelectric material[1,2], or due to the finite screening length in metallic electrodes field[3,4,5] Another example is the ferroelectric field-effect transistor[6], where the semiconducting layer causes an uncompensated depolarization field that limits data retention.

The presence of a dead layer leads to a large voltage offset along the horizontal (electric field) axis. The result is a deformed hysteresis loop with asymmetric switching characteristics. An

analytical bilayer model that describes the mechanism has been reported[7]. In our measurements the hysteresis loops are perfectly symmetrical and, hence, the presence of a dead layer can be disregarded.

Another reason for partial charge compensation is incomplete screening, for which there can be two causes. Firstly, the compensating charges in the electrode form a layer of finite thickness due to Thomas-Fermi screening length. Secondly, the polarization cannot drop abruptly when going from the ferroelectric to the metal, the so-called Kretschmer-Binder effect.

The effect of this depolarization field will become larger as the thickness of the ferroelectric decreases. The depolarization field is especially important in ultrathin films in the order of 10 nm, where it determines the critical thickness and domain structure.

For thick films the depolarization field from incomplete screening can be disregarded. Only when the ferroelectric is a perfect insulator, the incomplete screening leads to a finite depolarization field inside the ferroelectric material. However, due to the large film thickness this internal electric field is much smaller than the coercive field. Secondly, and more importantly, ferroelectric materials are not perfect insulators; tangent delta is finite and not zero. The uncompensated charges by the ferroelectric-electrode interface are neutralized by charge carriers in the ferroelectric material. Consequently, inside a thick film ferroelectric capacitor under short circuit conditions the internal electric field is zero. This statement is supported by the measured polarization of our samples, which is thickness independent. Furthermore, in ultrathin 15 nm $BaTiO_3$ films sandwiched between $SrRuO_3$ electrodes already 80 % of the remanent polarization is retained[8]. Finally, the remanent polarization of ultra-thin $PbTiO_3$ films saturates above 20 nm[9]. Therefore, in our electrostatic analysis the depolarization field due to incomplete screening in the electrodes can be disregarded.

Supplementary Note 3. Graphical extraction of the suppressed polarization

We connect a linear capacitor, C_{ser}, in series with a ferroelectric capacitor, C_{ferro}. At high bias the displacement is the same as for the ferroelectric-only capacitor, as then nearly all dipoles are aligned along the direction of the external electric field. The hysteresis loops have an identical apparent coercive voltage, independent of the ratio C_{ferro}/C_{ser}, as at zero displacement there are no net free charges in the electrodes and hence the equivalent circuit is that of a ferroelectric-only capacitor. Consequently, the hysteresis loops are tilted, and the remanent polarization is suppressed.

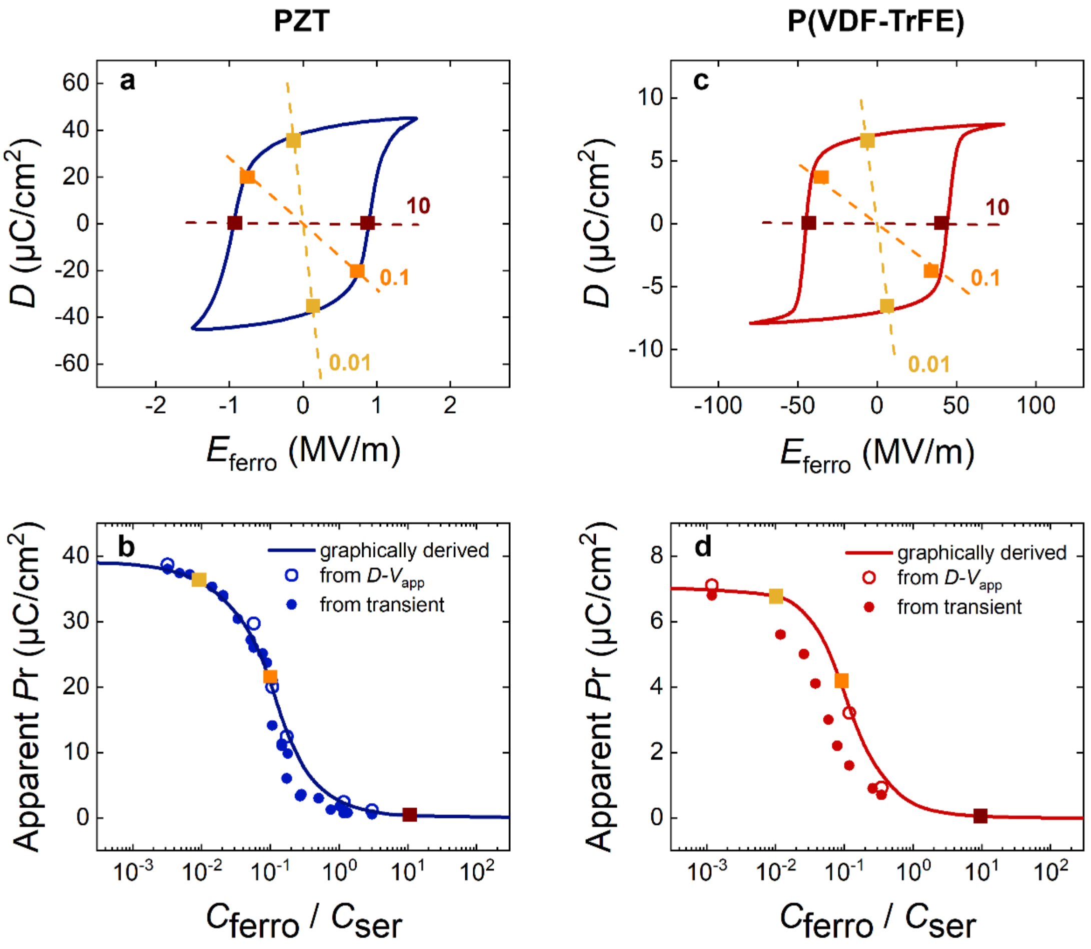

Supplementary Figure 1 | Suppression of polarization. (a,c) Graphical derivation of the apparent remanent polarization from the D-E_{ferro} hysteresis loop of ferroelectric-only capacitors of PZT and P(VDF-TrFE), respectively. The coloured dashed lines represent the linear relation between E_{ferro} and D at zero applied bias, calculated for three values of $C_{\mathrm{ferro}}/C_{\mathrm{ser}}$, as indicated. The displacement at the cross points with the hysteresis loops is by definition equal to the remanent polarization of the corresponding serial circuit. (b,d) Apparent remanent polarization, i.e. the displacement at zero applied voltage, as a function of the ratio of capacitances, $C_{\mathrm{ferro}}/C_{\mathrm{ser}}$, for (b) PZT and (d) P(VDF-TrFE) in serial circuits. The open circles are extracted from the quasi-static hysteresis loops. Solid circles are obtained from transient measurements. Solid lines present the graphically extracted values. The coloured squares correspond to the representative cross points in (a,c).

The value for the apparent remanent polarization can be graphically determined from the hysteresis D-E_{ferro} loop of the ferroelectric-only capacitor[10]. In a serial circuit the internal electric field in the ferroelectric material, E_{ferro}, is derived in the Methods section of the manuscript as:

$$E_{\text{ferro}} = \frac{V_{\text{app}}}{d} - \frac{D}{\varepsilon_0 \varepsilon_{\text{ferro}}} \cdot \frac{C_{\text{ferro}}}{C_{\text{ser}}} \tag{1}$$

where V_{app} is the applied bias and d is the thickness of the ferroelectric layer. At zero applied bias, the internal electric field is $E_{\text{ferro}} = -D \cdot (C_{\text{ferro}} / C_{\text{ser}})/(\varepsilon_0 \varepsilon_{\text{ferro}})$. For a given ratio of $C_{\text{ferro}}/C_{\text{ser}}$, this relation yields a straight line in the plot of D vs. E_{ferro}; its cross points with the D-E_{ferro} hysteresis loop of the ferroelectric-only capacitor determine the apparent remanent polarization, i.e. the electric displacement at zero applied bias, of the C_{ferro} and C_{ser} serial circuit. For a different value of $C_{\text{ferro}} / C_{\text{ser}}$, the line crosses the hysteresis loop at different points, as shown in Supplementary Figure 1a,c for PZT and P(VDF-TrFE) respectively. The coloured dashed lines represent the linear relation between E_{ferro} and D at zero applied bias for three representative values of $C_{\text{ferro}} / C_{\text{ser}}$. The cross points are marked with coloured squares.

The solid lines in Supplementary Figure 1b,d are the graphically extracted apparent remanent polarization as a function of the ratio $C_{\text{ferro}}/C_{\text{ser}}$. A good agreement with the apparent remanent polarization extracted from the quasi-static D-V_{app} hysteresis loops is obtained for both PZT and P(VDF-TrFE), which justifies our derivation of the internal field from the displacement using Supplementary Eq. 1.

Supplementary Note 4. Calculation of depolarization transients using the generalized KAI model

The switching of ferroelectric polarization is typically described by the model developed by Ishibashi[11], based on the classical statistical theory of nucleation and unrestricted domain growth, as described by Kolmogorov[12] and Avrami[13]. The change in ferroelectric polarization, $\Delta P(t)$, upon applying an electric field, E, is given by the compressed exponential function[26]:

$$\Delta P(t)/2P_{\text{r}} = 1 - exp\left[-\left(\frac{t}{t_0}\right)^n\right] \tag{2}$$

where t_0 is a characteristic switching time. In the conventional KAI approach, the Avrami index, n, depends on the dimensionality of the domains and takes only integer values: the value is 3 for single crystals and 2 for epitaxial thin films. However for polycrystalline thin films such as P(VDF-TrFE), the Avrami index is typically a non-integer between 1 and 2, depending on the strength of the electric field and the density of nucleation sites[32]. The switching time, t_0, follows the empirical Merz law[14]:

$$t_0 = t_\infty \cdot \exp\left[\frac{E_{\text{act}}(T)}{E}\right] \tag{3}$$

where E is the applied electric field, E_{act} is the so-called activation field[15] that is proportional to the domain-wall energy, and t_∞ is the switching time at infinite applied electric field. The Merz law is observed in many ferroelectric systems ranging from single crystals[14], through bulk ceramics[16], and thin films[17,18], to organic-ferroelectric composites[19]. We note that Tybel et al.[20] first pointed out that Merz law is a special case of domain-wall motion in generic creep systems, describing propagation of elastic objects driven by an external force in the presence of a pinning potential, such as domains in ferroelectric[20] and magnetic materials[21] and vortices in type-II superconductors[22]. For random-field type disordered ferroelectrics[20], the domain-wall creep velocity reads:

$$v \propto \frac{1}{t_0} \propto exp\left[-\frac{E_{act}(T)}{E}\right] \quad (4)$$

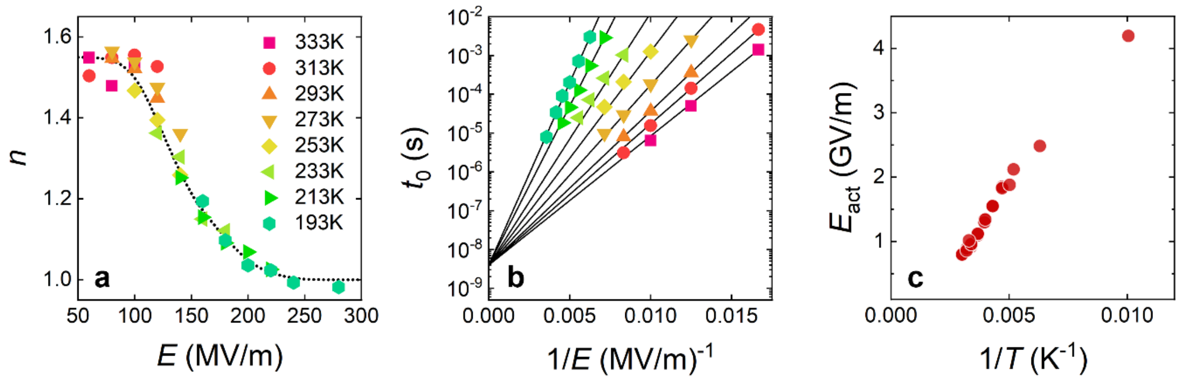

Supplementary Figure 2 | Switching parameters for P(VDF-TrFE). (a) The Avrami index, n, as a function of electric field. (b) The switching time, t_0, as a function of reciprocal electric field, and (c) the activation field, E_{act} as a function of reciprocal temperature. The values were extracted from macroscopic polarization reversal measurements of P(VDF-TrFE) capacitors at constant electric field[32,33] and reproduced from Ref.[32].

The KAI formalism has been previously adapted for a time-dependent electric field and used to calculate hysteresis loops as a function of ramping frequency[32]. Here we adapt the KAI formalism for a time-dependent depolarization field, $E_{dep}(t)$. After poling the ferroelectric material and removing the applied field, the internal field is derived as:

$$E_{ferro}(t) = E_{dep}(t)\frac{1}{1+(C_{ferro}/C_{ser})^{-1}} = -\frac{P(t)}{\varepsilon_0\varepsilon_{ferro}} \cdot \frac{1}{1+(C_{ferro}/C_{ser})^{-1}} \quad (5)$$

Supplementary Eq. 5 shows that the internal field and the polarization are mutually-dependent. Therefore both quantities need to be calculated iteratively. Depolarization transients were measured using a voltage pulse, high enough to fully polarize the ferroelectric capacitor. Then

the applied voltage abruptly dropped to 0 V, and we recorded the electric displacement as a function of time, $D(t)$. The iterative expression for the polarization reads:

$$P(t + \Delta t) = P(t) + [-P_{\mathrm{sat}} - P(t)] \cdot \left(1 - e^{-(t/t_0)^n}\right) \quad (6)$$

where the initial polarization is set equal to the fully reversed polarization, $-P_{\mathrm{sat}}$, as the internal field is opposite to the direction of the polarization. For each iteration the displacement, $D(t)$, is then calculated from:

$$D(t) = \varepsilon_0 \varepsilon_{\mathrm{ferro}} E_{\mathrm{ferro}}(t) + P(t) \quad (7)$$

The depolarization transient can then be calculated by iteratively solving Supplementary Eqs. 6 and 7. The parameters needed for the calculation are t_∞, n and E_{act} , all as a function of electric field. For P(VDF-TrFE) we take the values as reported previously[32,15], and which are reproduced in Supplementary Figure 2. Depolarization transients for PZT cannot yet be calculated as the full parameter set has not yet been reported.

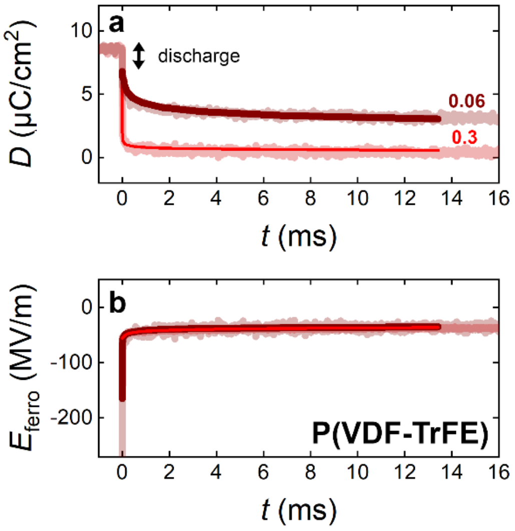

Supplementary Figure 3 | Depolarization dynamics for P(VDF-TrFE). (a) Displacement and (b) internal electric field as a function of time after applying a voltage pulse on a P(VDF-TrFE) capacitor in series with a linear capacitor. C_{ferro} of P(VDF-TrFE) here is 260 pF and the data correspond to serial capacitances of 4.4 nF and 890 pF, with the respective capacitance ratios indicated. The solid lines are obtained by iterative numerical calculation, viz. Supplementary Eqs.6 and 7.

The depolarization transients were measured at room temperature. We applied a voltage pulse, high enough to fully polarize the ferroelectric capacitor. Then the applied voltage abruptly dropped to 0 V as the electrodes were grounded, and the transient of the electric displacement was recorded. Typical transients for P(VDF-TrFE) for two values of $C_{\mathrm{ferro}}/C_{\mathrm{ser}}$ are presented in

Supplementary Figure 3. At the end of the applied pulse there is first a fast discharge of the induced polarization. Afterwards, the ferroelectric polarization dominates the transient. The solid lines in Supplementary Figure 3 are the calculated depolarization transients. The good agreement with the experimentally measured transients implies that depolarization is due to domain switching without macroscopic polarization reversal.

Supplementary Note 5. Negative differential capacitance (*NDC*)

During depolarization, both the internal electric field and the displacement are decreasing with time. However, as their signs are opposite, the depolarization transients feature a negative differential capacitance. An *NDC* as a transient phenomenon has been reported in ferroelectric capacitors during polarization reversal[23,24,25]. Here, the *NDC* is the derivative of the amount of free charges at the ferroelectric capacitor, Q_{ferro}, with internal voltage, V_{ferro}, and the absolute value is by definition equal to the serial capacitance, C_{ser}:

$$NDC = \frac{dQ_{\text{ferro}}}{dV_{\text{ferro}}} = \frac{A}{d} \cdot \frac{dD}{dE_{\text{ferro}}} = -C_{\text{ser}} \quad (8)$$

where we used Supplementary Eq. 1 for the relation between D and E_{ferro} at zero applied external bias. The *NDC* is constant during the whole depolarization process, unlike in the case of bipolar switching[23].

Supplementary Note 6. Temperature dependence of $P_r/\varepsilon_0\varepsilon_{\text{ferro}}E_c$ for P(VDF-TrFE)

To investigate the temperature dependence of $P_r/\varepsilon_0\varepsilon_{\text{ferro}}E_c$, we have measured the polarization, dielectric constant and the coercive field of P(VDF-TrFE) ferroelectric-only capacitors at temperatures between 213 K and 333 K.

The real part of the dielectric constant is presented as a function of frequency for various temperatures in Supplementary Figure 4a. The dielectric loss is measured to be 2% or less. The dielectric constant is almost frequency-independent below 10 kHz and slightly decreases at higher frequency. We take for the value of the static dielectric constant, $\varepsilon_{\text{ferro}}$, the dielectric constant at 1 kHz. Supplementary Figure 4a shows that the value of the static dielectric constant increases with temperature from 6 at 213 K to 11 at 333 K.

The temperature dependence of the coercive field, E_c, is presented in Supplementary Figure 4b. The values of E_c were extracted from quasi-static *D-E* hysteresis loops, measured in a Sawyer-Tower configuration at 100 Hz. As typical examples, hysteresis loops measured at three temperatures, viz. 213 K, 253 K and 333 K, are shown in the inset. The value of the coercive field monotonously decreases with increasing temperature from 115 MV/m at 213 K to 40

MV/m at 333 K. From the hysteresis loops we extracted the value of the remanent polarization, P_r, which slightly decreases from 8.8 μC/cm^2 at 213 K to 6.6 μC/cm^2 at 333 K. We note that the hysteresis loops indicate that the remanent polarization is almost equal to the saturated polarization; the difference in displacement between high bias and zero bias is dominated by the induced polarization, $\varepsilon_0 \varepsilon_{ferro} E$.

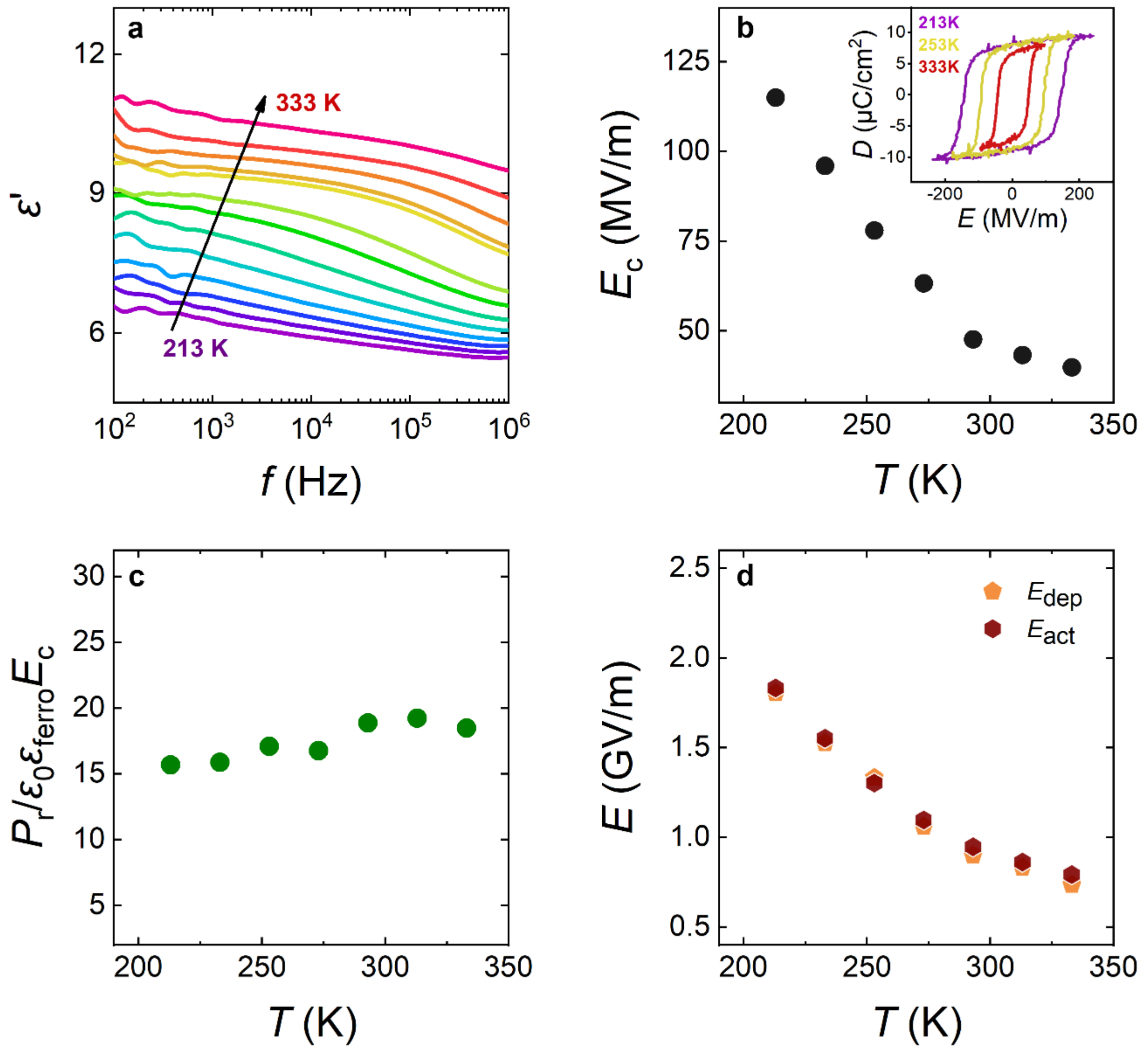

Supplementary Figure 4 | Temperature dependence of P(VDF-TrFE) ferroelectric-only capacitors. (a) The real part of the dielectric constant as a function of frequency at various temperatures between 213 K and 333 K. (b) The coercive field extracted from quasi-statically measured *D*-*E* hysteresis loops as a function of temperature. Hysteresis loops were measured at 100 Hz. Typical examples at three temperatures are shown in the inset. (c) Calculated value of $P_r/\varepsilon_0 \varepsilon_{ferro} E_c$ as a function of temperature. (d) The calculated depolarization field and the independently extracted activation field as a function of temperature. E_{dep} is calculated using the measured values of ε_{ferro} and P_r. E_{act} is extracted from polarization switching measurements[32,33].

The temperature dependence of the coercive field, E_c, is presented in Supplementary Figure 4b. The values of E_c were extracted from quasi-static *D-E* hysteresis loops, measured in a Sawyer-Tower configuration at 100 Hz. As typical examples, hysteresis loops measured at three temperatures, viz. 213 K, 253 K and 333 K, are shown in the inset. The value of the coercive field monotonously decreases with increasing temperature from 115 MV/m at 213 K to 40 MV/m at 333 K. From the hysteresis loops we extracted the value of the remanent polarization, P_r, which slightly decreases from 8.8 μC/cm^2 at 213 K to 6.6 μC/cm^2 at 333 K. We note that the hysteresis loops indicate that the remanent polarization is almost equal to the saturated polarization; the difference in displacement between high bias and zero bias is dominated by the induced polarization, $\varepsilon_0\varepsilon_{ferro}E$.

We calculated the value of $P_r/\varepsilon_0\varepsilon_{ferro}E_c$ using the extracted values of remanent polarization, static dielectric constant, and coercive field. As shown in Supplementary Figure 4c, the value is about 15 and independent of temperature between 213 K and 333 K. The constant value implies that at all temperatures the depolarization field, E_{dep}, is similar to the activation field, E_{act}. The temperature-dependent depolarization field, calculated from $P_r(T)/\varepsilon_0\varepsilon_{ferro}(T)$ is presented as a function of temperature in Supplementary Figure 4d, together with the activation field independently extracted from polarization switching measurements[32]. For all temperatures a perfect agreement is obtained, demonstrating that the relation E_{dep}~E_{act} holds for P(VDF-TrFE) within a wide range of temperatures.

Supplementary Note 7. Intrinsic switching

Intrinsic switching occurs at the intrinsic, or thermodynamic, coercive field $E_{int,c}$ where the Landau double potential well is destroyed. In the seminal work of Tagantsev et al., a relation between the intrinsic coercive field, the polarization P and the dielectric constant, has been theoretically derived as [Ref [26], Eq. 2.3.15, page 85]:

$$\frac{P}{\varepsilon_0\varepsilon_r E_{int,c}} = 3\sqrt{3} \approx 5 \qquad (9)$$

This relation is reminiscent of our experimental derived relationship, viz. $P_r/\varepsilon_0\varepsilon_{ferro}E_c \approx 15$. However, as will be explained below, the physical mechanisms are completely different, and cannot a priori be related.

Tagantsev et al. start with the mean-field treatment by Landau, where the Landau-Devonshire free energy, F, is expanded with an order parameter, the polarization P, as $F = -PE + \frac{1}{2}\alpha_0(T - T_0)P^2 + \frac{1}{4}\beta P^4$. Here P is the polarization, E is the electric field, α_0, β and T_0 are

Landau-Devonshire coefficients and T is the temperature. The stability condition, dF/dP = 0, leads to $E = \alpha_0(T - T_0)P + \beta P^3$. From this relation between E and P, the susceptibility is derived as $\chi_{\mathrm{LD}} = \frac{1}{2\varepsilon_0}\frac{\partial P}{\partial E} = \frac{1}{2\varepsilon_0 \alpha_0 (T_0 - T)}$ and the saturated polarization P_{LD} as $P_{LD} = \sqrt{\alpha_0(T_0 - T)/\beta}$ when taken $\left.\frac{\partial F}{\partial P}\right|_{E=0} = 0$. The magnitude of the intrinsic coercive field is the extreme value of E, as $\partial E/\partial P = 0$, and reads $E_{\mathrm{int,c}} = \frac{2}{3\sqrt{3}}\left(\frac{\alpha_0{}^3}{\beta}\right)^{1/2}(T_0 - T)^{3/2}$. By eliminating the Landau-Devonshire coefficients, the relation $\frac{P_{\mathrm{LD}}}{\varepsilon_0 \varepsilon_{\mathrm{LD}} E_{\mathrm{int,c}}} = 3\sqrt{3} \approx 5$ is obtained. Here we have taken the dielectric constant as $\varepsilon_{\mathrm{LD}} = \chi_{\mathrm{LD}} + 1 \approx \chi_{\mathrm{LD}}$.

Intrinsic switching is a mean-field process, which dictates collective behavior of the dipoles within the ferroelectric material. This happens when all dipoles are in a homogeneous environment; a necessary condition is that there are no pinning defects, which is practically unrealistic. Nevertheless, at very high electric field, the electrostatic energy dominates, thus influences from defects may become trivial: the pinning force by the defects is overwhelmed by the applied high field; the total electric field that the dipoles feel, either in defect-free regions or in vicinity of pinning defects, are about the same, and the system is effectively a homogeneous system. In that case, even a practical ferroelectric material with a considerable number of pinning defects may favour undergoing intrinsic switching.

Experimentally approaching the intrinsic switching has being a great challenge. To the best of our knowledge, experimental substantiation of intrinsic switching has only been reported in ultrathin films of $BaTiO_3$ and P(VDF-TrFE).

Ultrathin $BaTiO_3$(001) films were grown on a Pt(001)/MgO(001) substrate by laser ablation[27]. The coercive field was extracted from AFM measurements and reported as a function of layer thickness[28]. The coercive field increases with decreasing layer thickness. However, for thicknesses below about 10 nm the coercive field is constant, about 100 MV/m, therefore claimed to be the intrinsic coercive field. The depolarization field $P_{\mathrm{LD}}/\varepsilon_0\varepsilon_r E_{\mathrm{int,c}}$ was calculated by these authors to be about 200 MV/m [29] using a value of the dielectric constant of a single crystal $BaTiO_3$ of 150. This yields a value for $P_{\mathrm{LD}}/\varepsilon_0\varepsilon_{\mathrm{LD}}E_{\mathrm{int,c}} \sim 2$, in fair agreement with the theoretical value derived by Tagantsev et al. of about 5. Although this might suggest that $E_{\mathrm{dep}} \sim E_{\mathrm{int}}$, we note that $BaTiO_3$ is not a good model system as the dielectric constant varies over many orders of magnitude depending on chemical composition and microstructure. The dielectric constant of thin $BaTiO_3$ films also strongly depends on the film thickness[30]. To unambiguously correlate the depolarization and activation field with the intrinsic coercive field,

the comprising physical constants of the same device should be measured, which so far have not been reported.

P(VDF-TrFE) is an ideal model system as the dielectric constant hardly depends on microstructure and film thickness, and the complete data set on depolarization-, activation- and intrinsic coercive field is available. Intrinsic switching has been reported in Langmuir-Blodgett films of P(VDF-TrFE)[31]. The ferroelectric films with thickness between 1 nm and 10 nm are thin enough to inhibit nucleation. The intrinsic coercive field is independent of layer thickness and has been presented as a function of temperature. At ambient temperature $E_{\mathrm{int,c}}$ is measured to be about 600 MV/m. Taking the measured polarization as 0.1 C/m^2 and a dielectric constant of 10 then leads to a value for the depolarization field, $P_{\mathrm{LD}}/\varepsilon_0\varepsilon_r E_{\mathrm{int,c}}$, of about 1100 MV/m. The ratio $\frac{P_{\mathrm{LD}}}{\varepsilon_0 \varepsilon_{\mathrm{LD}} E_{\mathrm{int,c}}} \approx 2$ is in good agreement with the theoretical value derived by Tagantsev et al. of about 5.

Previously the experimentally extracted activation field of P(VDF-TrFE) as a function of temperature has been reported[32,33]. At ambient temperature a value of about 1000 MV/m was determined. This means that the activation field, depolarization field and intrinsic coercive field are of the same order of magnitude. Within a factor of two we arrive at $E_{\mathrm{dep}} \sim E_{\mathrm{act}} \sim E_{\mathrm{int,c}}$.

We note that this equality can be artificial as the underlying physical mechanisms for intrinsic and extrinsic switching are completely different. Current experiments, in our work as well as in literature, are insufficient to draw a solid conclusion about the relation with the intrinsic coercive field.

Supplementary References

1. Stengel, M. & Spaldin, N. A. Origin of the dielectric dead layer in nanoscale capacitors. *Nature* **443**, 679-682 (2006).

2. Stengel, M., Vanderbilt, D. & Spaldin, N. A. Enhancement of ferroelectricity at metal-oxide interfaces. *Nature Mater.* **8**, 392-397 (2009).

3. Mehta, R. R., Silverman, B. D. & Jacobs, J. T. Depolarization fields in thin ferroelectric films. *J. Appl. Phys.* **44**, 3379-3385 (1973).

4. Kim, D. J. et al. Polarization relaxation induced by a depolarization field in ultrathin ferroelectric BaTiO3 capacitors. *Phys. Rev. Lett.* **95**, 237602 (2005).

5. Gerra, G., Tagantsev, A. K., Setter, N. & Parlinski, K. Ionic polarizability of conductive metal oxides and critical thickness for ferroelectricity in BaTiO3. *Phys. Rev. Lett.* **96**, 107603 (2006).

6. Ma, T. P. & Han, J.-P. Why is nonvolatile ferroelectric memory field-effect transistor still elusive? *IEEE Electron. Device Lett.* **23**, 386-388 (2002).

7. Wong, C.K. & Shin, F.G. Modelling of an anomalous shift and asymmetric hysteresis behavior of ferroelectric thin films. *J. Appl. Phys.* **96**, 6648-6656 (2004)

8. Junquera, J. & Ghosez, P. Critical thickness for ferroelectricity in perovskite ultra-thin films. *Nature* **422**, 506-509 (2003).

9. Lichtensteiger, C., Triscone, J., Junquera J. & Ghosez, P. Ferroelectricity and tetragonality in ultrathin $PbTiO_3$ films. *Phys. Rev. Lett.* **94**, 047603 (2005).

10. Robels, U., Calderwood, J. H. & Arlt, G. Shift and deformation of the hysteresis curve of ferroelectrics by defects: an electrostatic model. *J. Appl. Phys.* **77**, 4002-4008 (1995).

11. Ishibashi, Y. & Takagi, Y. Note on ferroelectric domain switching. *J. Phys. Soc. Jpn.* **31**, 506-510 (1971).

12. Kolmogorov, A. On the statistical theory of the crystallization of metals. *Izv. Akad. Nauk USSR; Ser. Math.* **3**, 355-359 (1937).

13. Avrami, M. Kinetics of phase change. I general theory. *J. Chem. Phys.* **7**, 1103-1112 (1939).

14. Merz, W. J. Domain formation and domain wall motions in ferroelectric $BaTiO_3$ single crystals. *Phys. Rev.* **95**, 690-698 (1954).

15. Shin, Y.-H., Grinberg, I., Chen, I.-W. & Rappe, A. M. Nucleation and growth mechanism of ferroelectric domain-wall motion. *Nature* **449**, 881-884 (2007).

16. Zhukov, S. et al. Dynamics of polarization reversal in virgin and fatigued ferroelectric ceramics by inhomogeneous field mechanism. *Phys. Rev. B* **82**, 014109 (2010).

17. Gruverman, A., Wu, D. & Scott, J. F. Piezoresponse force microscopy studies of switching behavior of ferroelectric capacitors on a 100-ns time scale. *Phys. Rev. Lett.* **100**, 097601 (2008).

18. Jo, J. et al. Domain switching kinetics in disordered ferroelectric thin films. *Phys. Rev. Lett.* **99**, 267602 (2007).

19. Nautiyal, A. et al. Polarization switching properties of spray deposited $CsNO_3$: PVA composite films. *Appl. Phys. A* **99**, 941-946 (2010).

20. Tybell, T., Paruch, P., Giamarchi, T. & Triscone, J. Domain wall creep in epitaxial ferroelectric $Pb(Zr_{0.2}Ti_{0.8})O_3$ thin films. *Phys. Rev. Lett.* **89**, 097601 (2002).

21. Lemerle, S. et al. Domain wall creep in an Ising ultrathin magnetic film. *Phys. Rev. Lett.* **80**, 849-852 (1998).

22. Blatter, G., Feigel'man, M. V., Geshkenbein, V. B., Larkin, A. I. & Vinokur, V. M. Vortices in high-temperature superconductors. *Rev. Mod. Phys.* **66**, 1125-1388 (1994).

23. Khan, A. et al. Negative capacitance in a ferroelectric capacitor. *Nature Mater.* **14**, 182-186 (2015).

24. Jo, J. et al. Negative capacitance in organic/ferroelectric capacitor to implement steep switching MOS devices. *Nano Lett.* **15**, 4553-4556 (2015).

25. Zubko, P. et al. Negative capacitance in multidomain ferroelectric superlattices. *Nature* **534**, 524-528 (2016).

26. Tagantsev, A., Cross, L. & Fousek, J. Domains in Ferroic Crystals and Thin Films (Springer, 2010).

27. Gaynutdinov , R. et al. Polarization switching kinetics in ultrathin ferroelectric barium titanate film, *Physica B* **424**, 8-12 (2013).

28. Gaynutdinov , R. et al. Scaling of the Coercive Field in Ferroelectrics at the Nanoscale, *JETP Lett.* **98**, 339-341 (2013).

29. Fridkin, V.M. & Ducharme, S. General features of the intrinsic ferroelectric coercive field. *Phys. Solid State* **43**, 1320-1324 (2001).

30. Yano, Y., Iijima, K., Daitoh, Y., Terashima, T. & Bando, Y. Epitaxial growth and dielectric properties of BaTiO3 films on Pt electrodes by reactive evaporation. *J. App. Phys*. **76**, 7833-7838 (1994)

31. Ducharme, S. et al. Intrinsic Ferroelectric Coercive Field. *Phys. Rev. Lett.* **84**, 175-178 (2000).

32. Zhao, D., Katsouras, I., Asadi, K., Blom, P. W. M. & de Leeuw, D. M. Switching dynamics in ferroelectric P(VDF-TrFE) thin films. *Phys. Rev. B* **92**, 214115 (2015).

33. Hu, W. et al. Universal Ferroelectric Switching Dynamics of Vinylidene Fluoride-trifluoroethylene Copolymer Films. *Sci. Rep.* **4**, 4772 (2014).
